# Supplementary material for: Ultra-High-Capacity Lithium Metal Batteries Based on Multi-Electron Redox Reaction of Organopolysulfides including Conductive Organic Moieties
Source: Polymers (Basel). 2023 Jan 9;15(2):335. doi: 10.3390/polym15020335 (PMC9866748; doi:10.3390/polym15020335)
Supplement: Supplementary file 1 [file polymers-15-00335-s001.zip › polymers-1944711-supplementary.pdf]

polymers

MDPI

Supplementary Materials

Ultrahigh-capacity Lithium Metal Batteries Based on Multi-electron Redox Reaction of Organopolysulfides including Conductive Organic Moieties

Takeshi Shimizu *, Naoki Tanifuji *, Kosuke Nishio, Yuma Tanaka, Yuta Tsukaguchi, Kentaro Tsubouchi, Fumiya Nakamura, Naoko Shokura, Mariko Noguchi, Hiroki Fujimori, Hiromi Kimura-Suda, Yusuke Date, Kaoru Aoki, and Hirofumi Yoshikawa

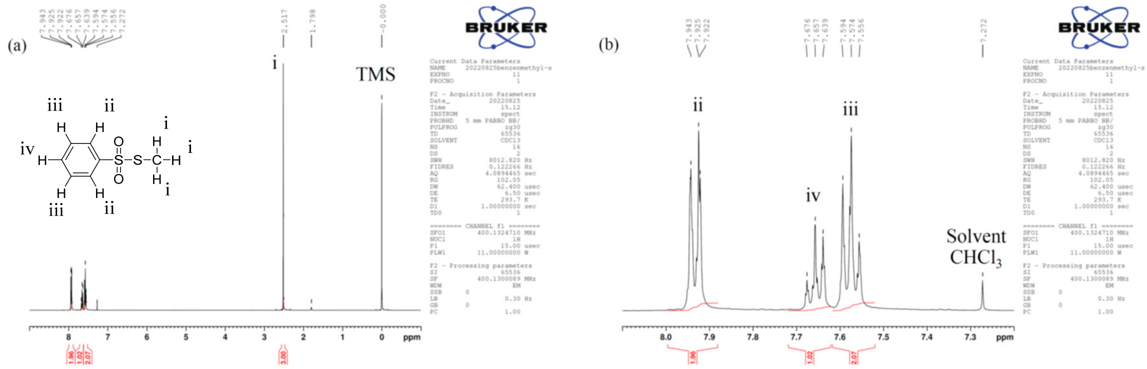

Figure S1. ^{1}H NMR charts of molecular **1**, 0-8 ppm (a) and 7.2-8.0 ppm (b).

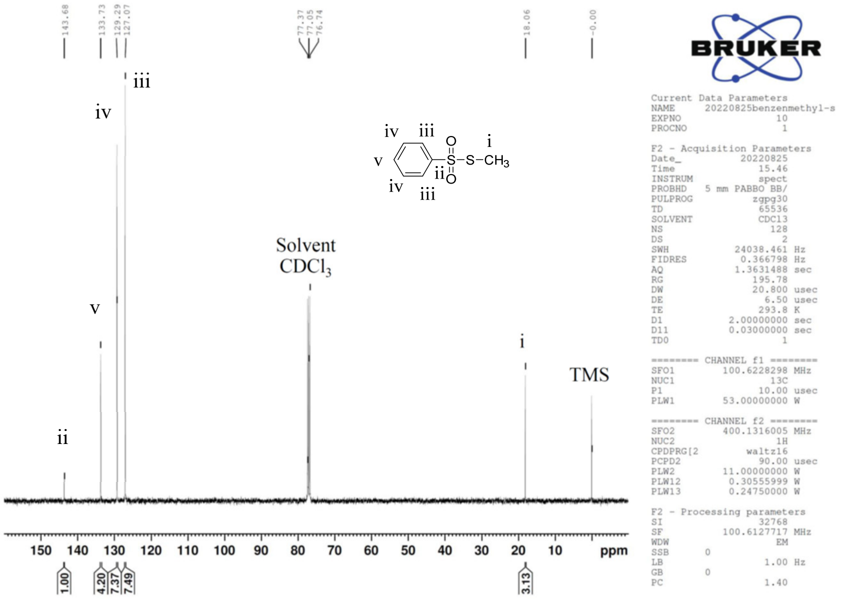

Figure S2. ^{13}C NMR chart of molecular **1**.

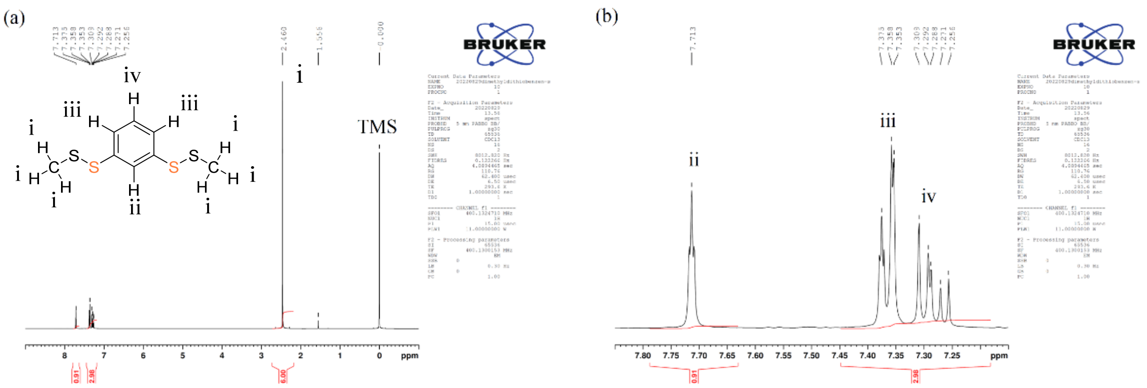

Figure S3. ^{1}H NMR charts of molecular **2a**, 0-8 ppm (a) and 7.15-7.85 ppm (b).

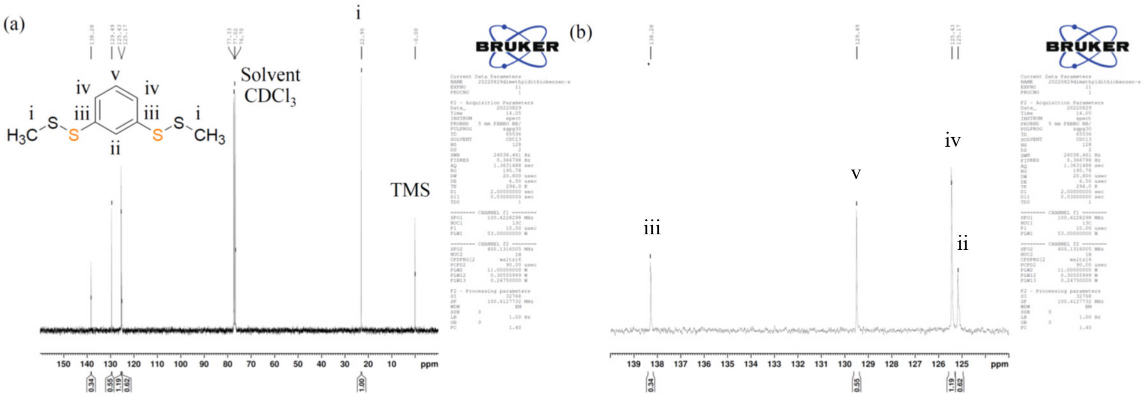

Figure S4. ^{13}C NMR charts of molecular **2a**, 0-160 ppm (a) and 123-140 ppm (b).

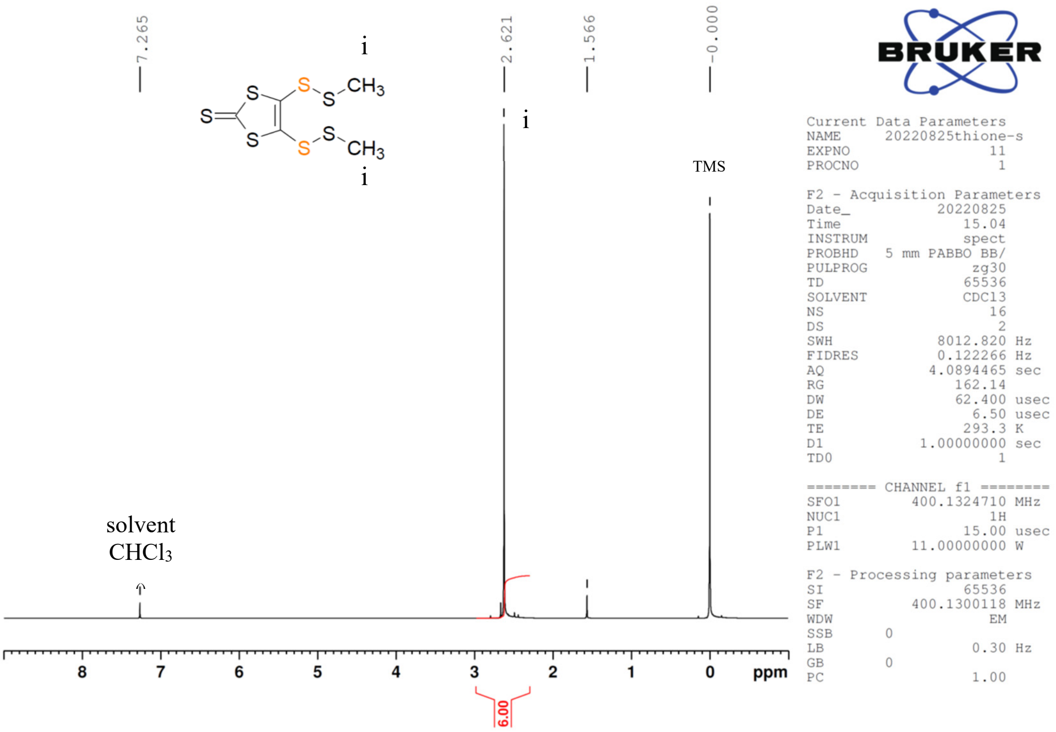

Figure S5. ^{1}H NMR chart of molecular **2b**.

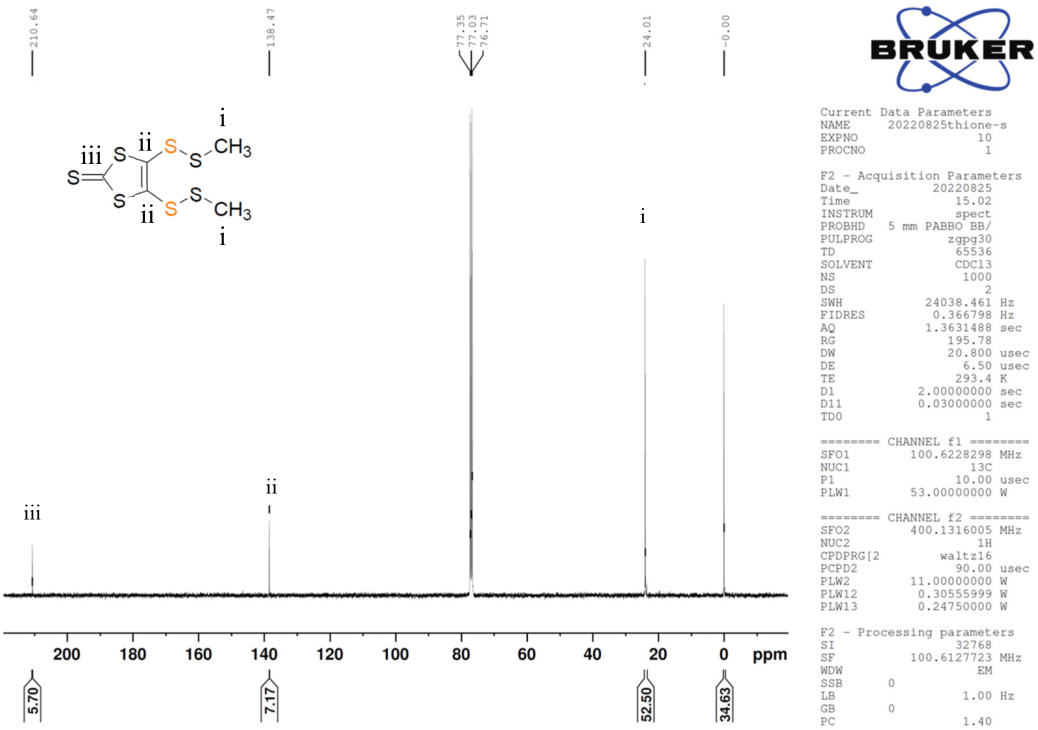

Figure S6. ^{13}C NMR chart of molecular **2b**.

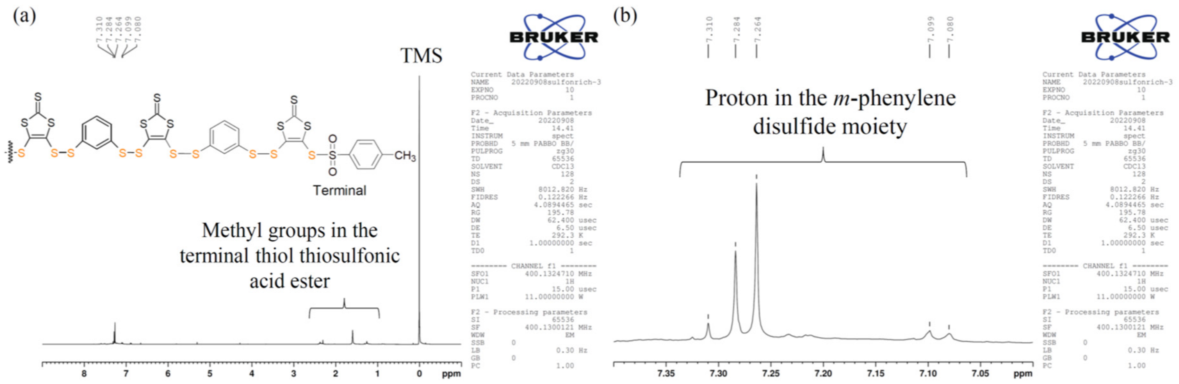

Figure S7. ^{1}H NMR chart of molecular **3’ab**, 0-8 ppm (a) and 7.0-7.4 ppm (b).

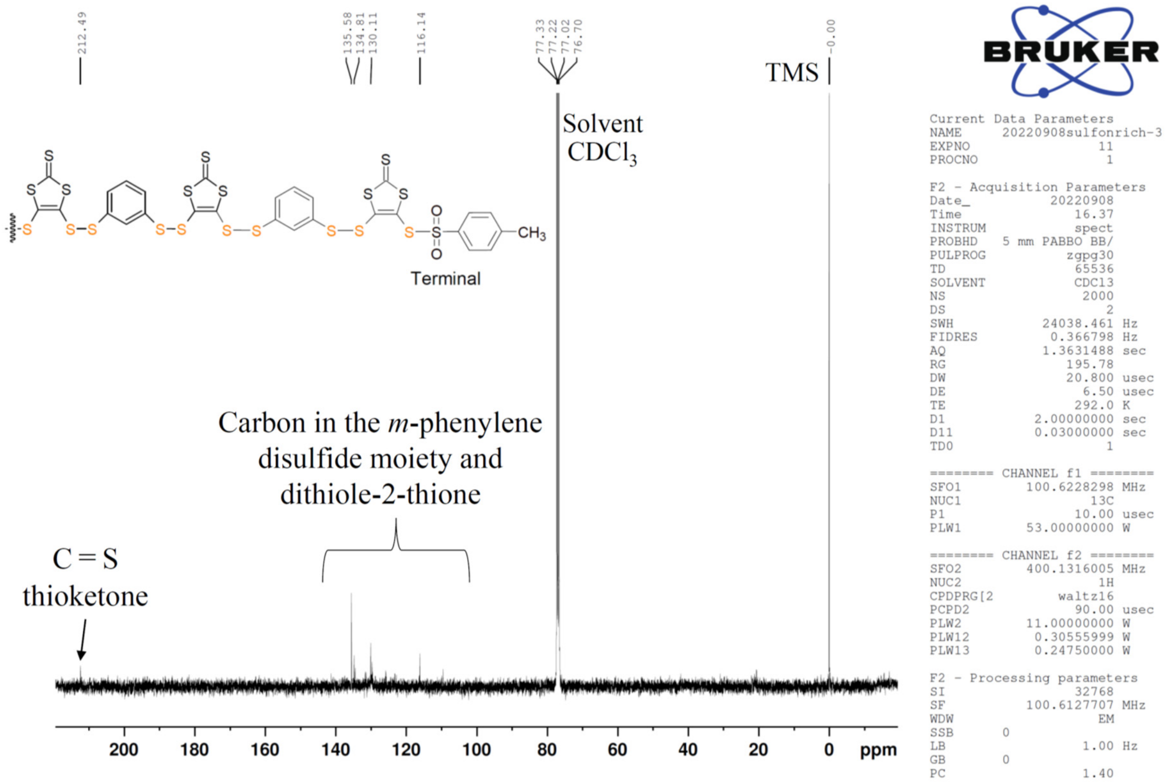

Figure S8. ^{13}C NMR chart of molecular **3’ab**.

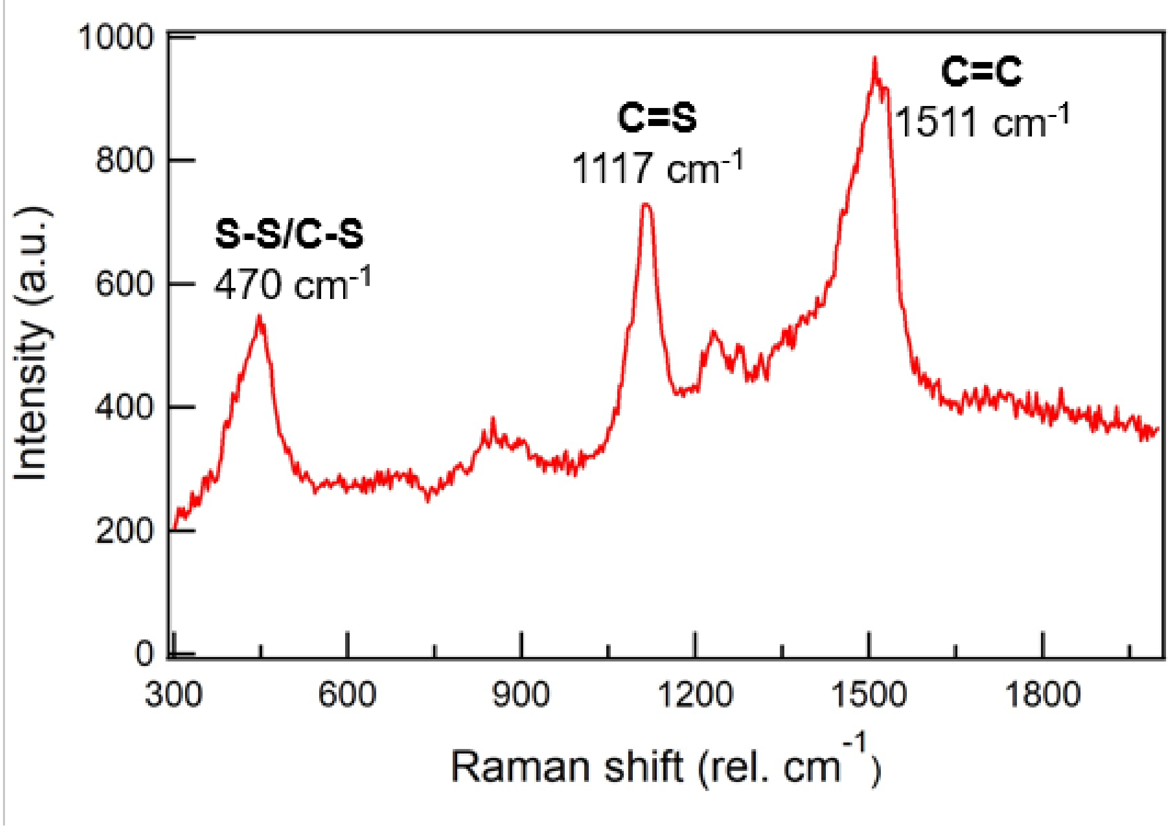

Figure S9. The Raman spectrum of **3ab**.

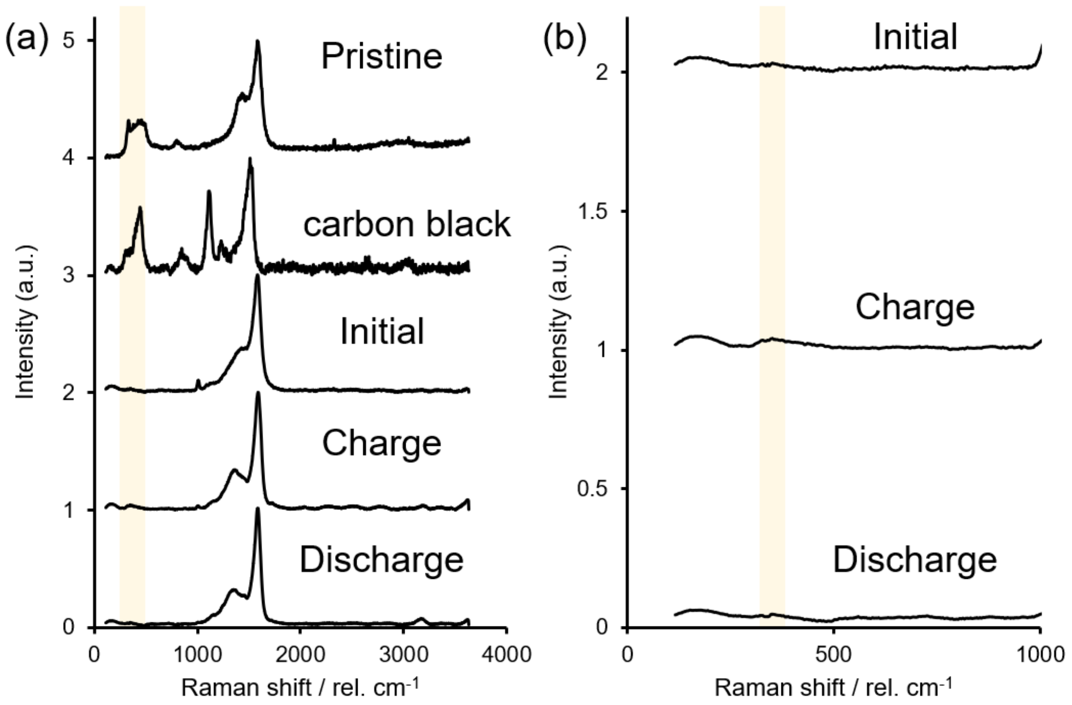

Figure S10. *Ex situ* Raman spectra of **3ab** in the initial, discharge, and charge states. (a) Raman shift: 0–4000 cm^{-1}, (b) 0–1000 cm^{-1}.

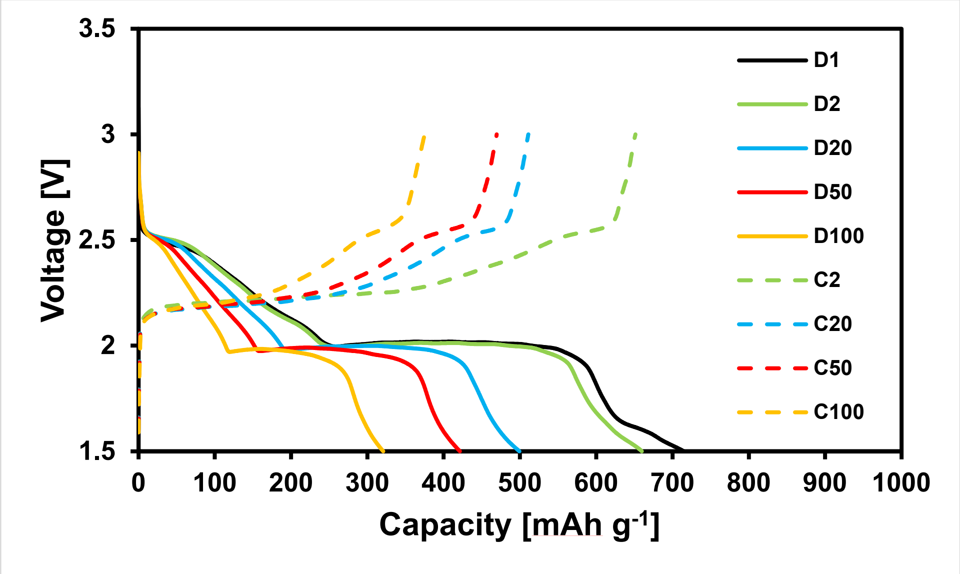

Figure S11. The discharge-charge curves of the battery including 70 wt% of **3ab** (carbon agent: 20 wt%, PVDF: 10 wt%).

Table S1. Comparison among the capacities of the conductive polymers with sulfur chains.

Samples	Current density [mA g^{-1}]	Initial capacity [mAh g^{-1}]	Capacity retention [mAh g^{-1}, cycles, %]	Ref
Polysulfide PPy	0.1 mA cm^{-2}	515	452, 20, 87.8	S1
Copolymer cp (S-g-PANI)/S	167.5	1002	733, 220, 73.2	S2
Capped CP(S3BT)	1675	928	682, 500, 73.5	S3
**3a**	200	750	445, 20, 59.3	**This work**
**3ab**	200	1096	681, 20, 62.1	**This work**

References

S1. Shi Chao, Z.; Lan, Z.; Jinhua, Y. Preparation and Electrochemical Properties of Polysulfide Polypyrrole. *J. Power Sources* **2011**, *196*, 10263–10266.

S2. Dai, S.; Feng, Y.; Wang, P.; Wang, H.; Liang, H.; Wang, R.; Linkov, V.; Ji, S. Highly Conductive Copolymer/Sulfur Composites with Covalently Grafted Polyaniline for Stable and Durable Lithium-Sulfur Batteries. *Electrochim. Acta* **2019**, *321*, 134678.

S3. Zeng, S.; Li, L.; Zhao, D.; Liu, J.; Niu, W.; Wang, N.; Chen, S. Polymer-Capped Sulfur Copolymers as Lithium-Sulfur Battery Cathode: Enhanced Performance by Combined Contributions of Physical and Chemical Confinements. *J. Phys. Chem. C* **2017**, *121* (5), 2495–2503.
